# Short-term clinical observations following local L8020 application in peri-implantitis with suppuration: a pilot study

**DOI:** 10.3389/froh.2026.1967259

**Published:** 2026-10-20

**Authors:** Yuhei Hashimoto, Keisuke Yasuda, Shinsuke Okada, Takashi Ogino, Kazuhiro Tsuga

**Affiliations:** Department of Advanced Prosthodontics, Hiroshima University Graduate School of Biomedical and Health Sciences, Hiroshima, Japan

**Keywords:** Lactobacillus rhamnosus KO3, microbiome, peri-implantitis, probiotics, suppuration

## Abstract

**Objectives:**

To evaluate feasibility, short-term safety, and preliminary clinical observations following local application of Lactobacillus rhamnosus KO3 (L8020) after standardized saline irrigation in patients with peri-implantitis presenting with suppuration.

**Materials and methods:**

This prospective, single-arm, open-label pilot clinical study enrolled ten patients with peri-implantitis presenting with suppuration. After standardized irrigation of the peri-implant sulcus, L8020 was topically applied. Clinical examinations and submucosal microbiological sampling were performed at baseline and Day 7. The primary outcome was disappearance of suppuration at Day 7. The prespecified secondary outcome was change in the peri-implant microbial community; PPD was exploratory, and adverse events were monitored. Microbiome analyses included 16S rRNA V3-V4 sequencing, paired alpha-diversity analysis, Bray-Curtis beta-diversity analysis, taxonomic composition profiling, and exploratory paired differential-abundance testing.

**Results:**

Suppuration was absent in 7 of 10 implants (70%) at Day 7, whereas mean PPD remained unchanged (7.1 ± 1.9 mm at both time points). No significant changes were detected in five alpha-diversity indices. Bray-Curtis PCoA showed no distinct separation between baseline and Day 7, and a paired PERMANOVA using all 2^10 within-participant label permutations showed no difference in overall community structure (pseudo-F = 0.158; exact *p* = 0.883). Genus- and species-level taxonomic profiles showed no consistent community-wide shift, and no genus or species remained significant after paired Wilcoxon testing with Benjamini-Hochberg correction. No intervention-related adverse events were observed.

**Conclusions:**

The combined local procedure was feasible and well tolerated over 7 days. The disappearance of suppuration is a preliminary short-term observation and should not be interpreted as resolution of peri-implantitis or evidence of an L8020-specific therapeutic effect. The clinical observation occurred without a detectable major compositional shift in the peri-implant microbiota. Controlled studies with an irrigation-only comparator, larger samples, and longer follow-up are required.

**Clinical Trial Registration:**

https://jrct.mhlw.go.jp/latest-detail/jRCTs061230059, Japan Registry of Clinical Trials (jRCT) jRCTs061230059.

## Introduction

Peri-implantitis is one of the most important biological complications associated with dental implant therapy and is characterized by inflammation of peri-implant tissues accompanied by progressive bone loss. As the use of dental implants continues to increase worldwide, the number of patients affected by peri-implant diseases is expected to rise accordingly. Recent systematic reviews have estimated the prevalence of peri-implantitis to be approximately 20%–25%, although reported values vary depending on the diagnostic criteria applied (1, 2). Consequently, peri-implantitis has become a major challenge in contemporary implant dentistry.

Peri-implantitis is a biofilm-associated inflammatory disease in which dysbiosis of the peri-implant microbiota and an exaggerated host inflammatory response contribute to progressive tissue destruction (3). Clinically, suppuration is regarded as an important sign of active inflammation and frequently motivates patients to seek treatment. Compared with periodontal tissues surrounding natural teeth, peri-implant tissues differ in anatomical structure, vascular supply, and healing capacity, potentially resulting in more rapid disease progression and less predictable treatment outcomes (4).

Although numerous nonsurgical and surgical treatment modalities have been proposed, management of peri-implantitis remains challenging (5, 6). Mechanical debridement remains central to nonsurgical therapy; however, complete decontamination of implant surfaces is often difficult because of implant thread geometry and surface roughness. Adjunctive approaches, including antiseptics, systemic or local antibiotics, lasers, photodynamic therapy, and air-polishing devices, have been investigated, yet no single intervention has consistently demonstrated superior outcomes. These limitations have stimulated interest in approaches intended to modify the peri-implant microbial environment.

Probiotics, defined as live microorganisms that confer a health benefit on the host when administered in adequate amounts (7), have attracted attention as adjunctive strategies for biofilm-associated oral diseases. Clinical studies have suggested that probiotics may provide additional benefits when combined with conventional periodontal therapy; however, the magnitude and consistency of these effects remain uncertain (8, 9). Evidence regarding probiotics for peri-implantitis remains limited.

We previously reported that implants with peri-implantitis in remission exhibited higher relative abundances of lactic acid bacteria, including Lactobacillales and Bifidobacterium, than implants with active suppuration (10). These observations provided a biological rationale for exploring probiotic approaches. Lactobacillus rhamnosus KO3 (L8020) has been reported to inhibit cariogenic and periodontopathogenic bacteria and to interfere with biofilm formation (11). However, no clinical study has evaluated topical L8020 application in patients with peri-implantitis.

The present prospective pilot study therefore evaluated the feasibility, short-term safety, and preliminary clinical observations following topical L8020 application after standardized saline irrigation in patients with peri-implantitis presenting with suppuration. Changes in the peri-implant microbiota were prespecified as an exploratory secondary component and were examined using 16S rRNA gene sequencing to characterize short-term community-level and taxonomic changes rather than to establish microbiological efficacy or mechanism.

## Materials and methods

### Study design and ethical approval

This prospective, single-arm, open-label pilot clinical study was conducted in accordance with the Declaration of Helsinki (12) and the Japanese Clinical Trials Act. The study protocol was approved by the Ethics Committee for Clinical Research of Hiroshima University (Certified Review Board No. CRB6180006). The trial was registered in the Japan Registry of Clinical Trials (jRCT; jRCTs061230059). Written informed consent was obtained from all participants before enrollment. During the study period, an administrative non-compliance occurred during transition of the principal investigator, resulting in enrollment of three participants while the position of principal investigator was temporarily vacant. The incident was formally reported and reviewed through institutional procedures, corrective and preventive measures were implemented, and the data from these three participants were permitted to be included in the analysis.

### Participants

Patients who visited the participating clinic between 2023 and 2025 and were diagnosed with peri-implantitis presenting with suppuration were screened for eligibility. Ten implants from ten patients meeting the inclusion criteria were enrolled. Peri-implantitis was diagnosed based on peri-implant inflammation, radiographic evidence of progressive bone loss, and suppuration on gentle probing. When multiple implants fulfilled the eligibility criteria, the implant with the longest documented duration of suppuration was selected as the target implant.

The inclusion criteria were: (1) age ≥18 years; (2) provision of written informed consent; (3) at least one implant diagnosed with peri-implantitis for ≥1 year; and (4) peri-implantitis with persistent suppuration for at least 3 months, confirmed within 1 week before enrollment. Exclusion criteria were: (1) use of products containing lactic acid bacteria, bifidobacteria, butyric acid bacteria, or L8020 within 3 months before enrollment; (2) a history of severe adverse reactions to probiotic preparations or the investigational product; (3) use of topical or systemic antimicrobial agents within 7 days before enrollment; and (4) current use of L8020. Systemic conditions were recorded using a standardized medical questionnaire but were not used as exclusion criteria unless they met predefined criteria. None of the participants were current smokers. All participants had previously received oral hygiene instruction and demonstrated a plaque control record <20% at enrollment. Although periodontal pockets ≥4 mm were present at some sites, periodontal conditions were clinically stable under supportive periodontal care. All participants had received supportive implant maintenance at 3–6-month intervals before study entry.

### Intervention

The investigational product was a gel containing 2.5% Lactobacillus rhamnosus KO3 (L8020) (JEX Co., Ltd., Hiroshima, Japan). Each 80 g of product contained 2 g of L8020 together with dipotassium glycyrrhizinate, potassium nitrate, sodium fluoride, and sodium polyphosphate. Before application, the peri-implant sulcus was gently irrigated with 2.5 mL of sterile saline for approximately 10 s to remove visible suppuration; the same irrigation procedure was used for all participants. Approximately 0.3 mL of L8020 was then applied circumferentially into the peri-implant sulcus using a syringe designed for subgingival administration until slight overflow of gel was visually observed. Participants were instructed not to eat, drink, gargle, or use antimicrobial mouthwash for 30 min after application. Systemic or topical antimicrobial agents were prohibited during the 7-day observation period. Participants continued usual toothbrushing, and no additional nonsurgical or surgical treatment was performed at the target implant.

### Clinical examination

Clinical examinations were performed by a single experienced examiner (Y.H.). A plastic Williams Colorvue periodontal probe (Hu-Friedy Mfg. Co., LLC, Chicago, IL, USA) was used for all measurements. Probing was performed with a gentle force of approximately 0.25 N. PPD was measured at six sites around each implant, and the deepest PPD was used for statistical analysis. Bleeding on probing (BOP) and suppuration were assessed at the same six sites. Suppuration was considered present when visible purulent exudate was observed at one or more sites after gentle probing. Because this was a single-arm pilot study, the examiner was not blinded to the intervention.

### Microbiological sampling, sequencing, and bioinformatic analysis

Before clinical examination or probing, submucosal plaque samples were collected from the deepest peri-implant sulcus using sterile paper points inserted for approximately 30 s and immediately transferred to sterile collection tubes. Sampling was performed at baseline and Day 7. Clinical examination was performed after sampling to minimize disturbance of the submucosal biofilm before collection.

Microbiological processing and sequencing were performed by Seibutsu Giken Inc. (Kanagawa, Japan). Samples were mechanically disrupted after addition of Lysis Solution F (Nippon Gene, Japan) using a Shake Master Neo (bms) at 1,500 rpm for 2 min. Samples were then incubated at 65 °C for 10 min and centrifuged at 12,000 × g for 2 min. DNA was purified from the recovered supernatant using the Lab-Aid824s DNA Extraction Kit (ZEESAN). DNA concentration was measured using the QuantiFluor dsDNA System (Promega).

The V3-V4 region of the bacterial 16S rRNA gene was amplified using a two-step tailed PCR approach. Mixed 341F/805R-based primers incorporating 0–5-base random sequences were used for the first PCR. Libraries were sequenced on an Illumina NextSeq 1000 system using NextSeq 1000/2000 P1 Reagents (600 cycles) with 2 × 300-bp paired-end reads.

Sequence preprocessing used FASTX-Toolkit version 0.0.14 to retain reads matching the primer sequences and remove primer sequences, followed by Sickle version 1.33 for quality filtering. Reads with quality scores <20 were removed, and reads shorter than 130 bases and their paired reads were discarded. Paired-end reads were merged using FLASH version 1.2.11. Subsequent analysis was performed using QIIME2 version 2024.10. The DADA2 plugin was used to remove chimeric and noise sequences and generate representative sequences and an amplicon sequence variant (ASV) table. Taxonomic assignment was performed using the QIIME2 feature-classifier plugin against the Human Oral Microbiome Database (HOMD), version 15.23. Alignment and phylogeny plugins were used for phylogenetic analyses.

Taxonomic composition was summarized at multiple levels, including genus and species. Alpha diversity was evaluated using Chao1, Faith's phylogenetic diversity (Faith's PD), observed features, Pielou evenness, and Shannon diversity. For alpha-diversity comparisons, values at 34,677 reads, the highest rarefaction depth available for all 20 samples, were averaged across 10 rarefaction iterations for each sample and compared between baseline and Day 7 using paired Wilcoxon signed-rank tests. Beta diversity was examined using Bray-Curtis distances and visualized by principal coordinate analysis (PCoA). To account for the paired design, overall community structure was compared using an exact paired PERMANOVA in which baseline/Day 7 labels were exchanged only within each participant; all 2^10 = 1,024 possible within-participant label permutations were evaluated. Exploratory paired differential-abundance analyses at genus and species levels used Wilcoxon signed-rank tests with Benjamini-Hochberg false-discovery-rate correction. The original contract-laboratory LEfSe analysis (version 1.0.8) across phylum through species levels was retained as a complementary exploratory analysis. Because of the small sample size, all microbiome analyses were interpreted as exploratory rather than confirmatory evidence of microbiological efficacy. ChatGPT (OpenAI; GPT-5.6 Sol and GPT-6) was used to assist with the planning, coding, computational implementation, and visualization of the supplementary exploratory paired microbiome analyses described above. The statistical methods, input data, and interpretation of the results remain the responsibility of the authors.

### Outcome measures

The primary outcome was disappearance of suppuration at Day 7. This prespecified registry outcome is reported as a short-term clinical observation; absence of visible suppuration at Day 7 does not imply resolution or sustained remission of peri-implantitis. The prespecified secondary outcome was change in the peri-implant microbial community. PPD was additionally assessed as an exploratory clinical measure, and adverse events were monitored as part of the safety assessment. A 7-day follow-up was selected to investigate early clinical and microbiological changes after the local procedure; it was not intended to establish sustained efficacy.

### Safety assessment

Adverse events were assessed throughout the study period by clinical examination and patient interviews. Local adverse reactions, including pain, swelling, mucosal irritation, allergic reactions, and unexpected oral findings, as well as systemic adverse events, were recorded and evaluated for possible relationship to the investigational product.

### Statistical analysis

No formal sample size calculation was performed because no previous clinical studies evaluating topical L8020 for peri-implantitis were available. The target sample size of 10 implants was determined pragmatically based on feasibility and the exploratory nature of the pilot study. Clinical outcomes were analyzed descriptively. Disappearance of suppuration was expressed as the proportion of implants without suppuration at Day 7, and continuous clinical variables are presented as mean ± standard deviation. Microbiome comparisons respected the paired baseline/Day 7 design. Alpha-diversity indices were compared using paired Wilcoxon signed-rank tests. Bray-Curtis beta diversity was evaluated with exact paired PERMANOVA using all 1,024 possible within-participant label permutations. Taxon-wise paired Wilcoxon tests with Benjamini-Hochberg correction were used as exploratory differential-abundance analyses at genus and species levels. All tests were two-sided, and microbiome findings were interpreted cautiously because of the small sample size and exploratory design.

## Results

### Study population

Ten eligible participants (10 implants) were enrolled, all received the study intervention, and all completed the Day 7 follow-up. No missing clinical or microbiological data were observed. Participant flow is shown in Figure 1, and baseline demographic and clinical characteristics are summarized in Table 1. All target implants fulfilled the predefined diagnostic criteria for peri-implantitis and exhibited persistent suppuration at baseline.

**Figure 1:**
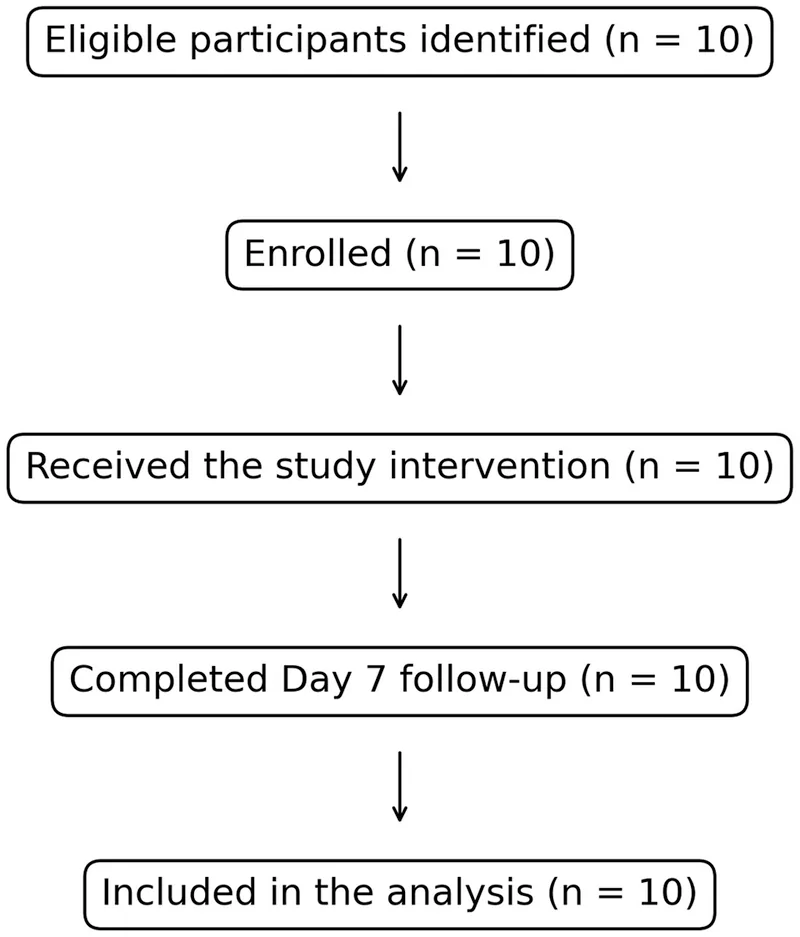
Participant flow through the study: eligible participants identified (*n* = 10), enrolled (*n* = 10), received the study intervention (*n* = 10), completed day 7 follow-up (*n* = 10), and included in the analysis (*n* = 10).

**Table 1:** Baseline demographic and clinical characteristics of the study participants.

Characteristic	Value
Number of participants	10
Number of implants	10
Age (years), mean ± SD	80.0 ± 7.8
Sex, *n* (%)	Male, 3 (30%); Female, 7 (70%)
Medical history, *n* (%)	1 (10%)
History of cerebral infarction	1 (10%)
Time in function	≥1 year (all implants)
Duration of suppuration	≥3 months (all implants)
PPD (mm), mean ± SD	7.1 ± 1.9
BOP, *n* (%)	10 (100%)
Suppuration, *n* (%)	10 (100%)
Supportive implant maintenance interval	Every 3–6 months

Values are presented as mean ± standard deviation (SD) or number (%), unless otherwise indicated. PPD, probing pocket depth; BOP, bleeding on probing.

### Clinical outcomes

At Day 7, suppuration was absent in 7 of the 10 implants (70%) and remained present in the other three implants. Mean PPD was 7.1 ± 1.9 mm at baseline and remained 7.1 ± 1.9 mm at Day 7; no PPD changes were observed in any participant. Individual clinical findings are summarized in Table 2.

**Table 2:** Clinical findings at baseline and day 7 following topical application of Lactobacillus rhamnosus KO3 (L8020).

Case	PPD (mm)		BOP		Suppuration	
	Baseline	Day 7	Baseline	Day 7	Baseline	Day 7
1	10	10	+	−	+	−
2	5	5	+	−	+	−
3	4	4	+	−	+	−
4	5	5	+	−	+	−
5	8	8	+	+	+	+
6	6	6	+	−	+	−
7	8	8	+	+	+	−
8	9	9	+	−	+	+
9	8	8	+	−	+	−
10	8	8	+	−	+	+

PPD values were unchanged between baseline and Day 7 in all participants. PPD, probing pocket depth; BOP, bleeding on probing.

### Microbiological outcomes

Submucosal plaque samples obtained at baseline and Day 7 were successfully analyzed in all participants. None of the five alpha-diversity indices showed a significant paired change between baseline and Day 7 (Chao1, *p* = 0.557; Faith's PD, *p* = 0.557; observed features, *p* = 0.557; Pielou evenness, *p* = 0.695; Shannon diversity, *p* = 0.770; Supplementary Table S1). Bray-Curtis PCoA showed no distinct separation between baseline and Day 7 microbial communities (Figure 2A). Exact paired PERMANOVA, restricted to within-participant label exchanges, likewise showed no difference in overall community structure (pseudo-F = 0.158; exact *p* = 0.883; 1,024 permutations).

**Figure 2:**
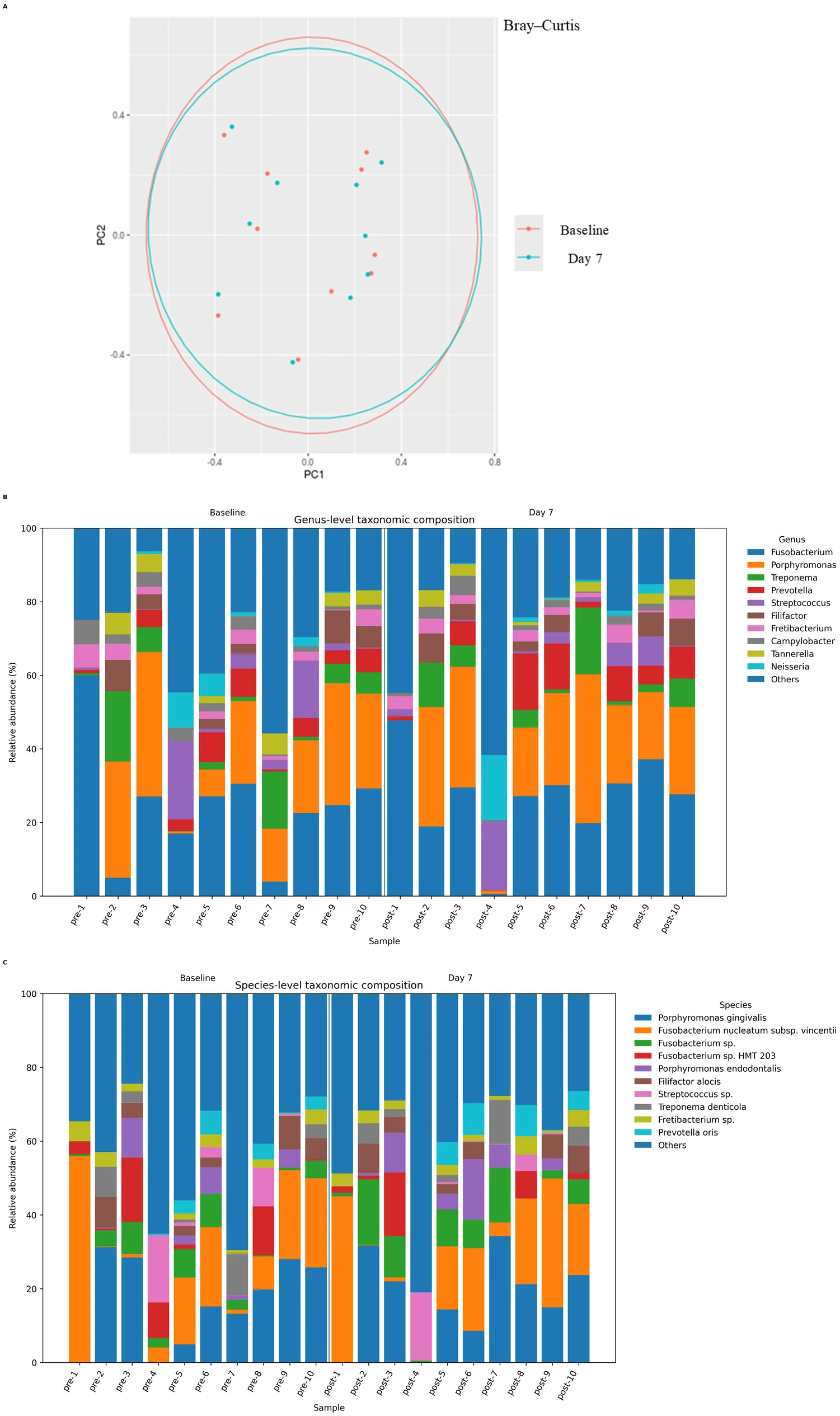
Microbiome analyses at baseline and day 7. **(A)** Principal coordinate analysis (PCoA) based on Bray-Curtis distances. No distinct separation between baseline and Day 7 samples was observed; exact paired PERMANOVA using all 1,024 within-participant label permutations showed no difference in overall community structure (pseudo-F = 0.158; exact *p* = 0.883). **(B)** Genus-level relative taxonomic composition for individual baseline and Day 7 samples. **(C)** Species-level relative taxonomic composition for individual baseline and Day 7 samples. Panels B and C show the nine most abundant taxa across all samples, with remaining taxa grouped as others.

Genus- and species-level taxonomic composition profiles showed substantial inter-individual variation without a consistent community-wide shift from baseline to Day 7 (Figures 2B,C). Across all samples, Fusobacterium and Porphyromonas were the most abundant genera. At the species level, Porphyromonas gingivalis and Fusobacterium nucleatum subsp. vincentii were among the most abundant taxa at both time points. Exploratory paired taxon-wise Wilcoxon analyses identified no genus or species that remained significant after Benjamini-Hochberg correction (120 genera and 290 species tested; minimum adjusted q = 1.000). The original LEfSe analysis likewise identified no significant differential abundance between baseline and Day 7 from phylum through species level. Taken together, the alpha-diversity, paired beta-diversity, taxonomic-profile, and differential-abundance analyses did not demonstrate a major compositional restructuring of the peri-implant microbiota during the 7-day observation period.

### Safety outcomes

No intervention-related adverse events were observed. No participants experienced local irritation, swelling, allergic reactions, or other oral complications attributable to topical L8020, and no systemic adverse events considered related to the investigational product were reported.

## Discussion

In this prospective pilot clinical study, topical L8020 application following standardized saline irrigation was feasible and well tolerated, and suppuration was absent at Day 7 in 70% of implants. No intervention-related adverse events occurred. However, because this was an uncontrolled single-arm study and saline irrigation and local manipulation were performed immediately before L8020 application, the observed short-term change cannot be attributed specifically to L8020. The study therefore provides information on feasibility, short-term safety, and preliminary clinical observations rather than therapeutic efficacy.

No measurable change in PPD was observed over 7 days. Given the very short follow-up, the absence of PPD change is not unexpected; importantly, it also demonstrates that the observed clinical change was restricted to the sign of suppuration and was not accompanied by measurable structural clinical improvement. The Day 7 observation should therefore not be equated with resolution of peri-implantitis.

The expanded microbiome analyses likewise did not identify a substantial short-term community shift. None of the five alpha-diversity indices changed significantly. Bray-Curtis PCoA showed no distinct separation between time points, and exact paired PERMANOVA did not detect a difference in overall community structure. Genus- and species-level taxonomic profiles showed marked inter-individual variation but no consistent restructuring from baseline to Day 7. Paired taxon-wise analyses with false-discovery-rate correction identified no differentially abundant genus or species, and the complementary LEfSe analysis was also negative from phylum through species. These convergent findings indicate that the short-term clinical observation was not accompanied by a detectable major change in bacterial community composition.

This negative microbiome finding should be interpreted cautiously. 16S rRNA gene sequencing characterizes relative bacterial composition but does not directly measure bacterial viability, metabolic activity, host inflammatory responses, or strain-specific persistence of L8020. The study also did not use a strain-specific molecular method to confirm colonization or persistence of L8020. Therefore, the present results neither establish nor exclude transient functional or host-mediated effects that might occur without a detectable compositional shift. Conversely, the disappearance of suppuration cannot be interpreted as evidence that L8020 altered the microbiome or mediated the clinical change.

Because standardized saline irrigation and direct manipulation of the peri-implant sulcus were performed immediately before L8020 application, multiple alternative explanations remain plausible. Irrigation, physical disturbance of local biofilm, removal or dilution of inflammatory exudate, modification of the local wound environment, and spontaneous short-term fluctuation in disease activity could each contribute to temporary disappearance of suppuration. Accordingly, the Day 7 finding is best interpreted as a possible transient remission of suppuration after a combined local procedure rather than as evidence of a probiotic-specific treatment effect.

The findings can also be considered in the context of the TIME wound-healing model discussed by Miranda-Rius et al. (13), in which active intervention in the local wound environment, disruption or removal of detrimental biofilm and inflammatory material, repeated reassessment, and patient self-care are used to restore balance and foster repair. In the present study, saline irrigation and direct sulcular manipulation may themselves have altered the local environment independently of any L8020-specific effect. The procedure evaluated here therefore represents a limited local intervention and should not be regarded as comprehensive or definitive peri-implantitis therapy.

Several limitations are fundamental to interpretation. First, this was a single-center study with only 10 participants. Second, the single-arm design without an irrigation-only or placebo control prevents causal inference and makes it impossible to separate any L8020-specific effect from irrigation, local manipulation, biofilm disruption, spontaneous fluctuation, or other nonspecific factors. Third, follow-up was limited to 7 days, so disappearance of suppuration may represent only transient remission and cannot be interpreted as sustained treatment success. Fourth, neither PPD improvement nor a substantial microbiome shift was demonstrated. Finally, strain-specific persistence of L8020 was not evaluated. These design constraints limit the reliability and generalizability of any efficacy inference and are the reason the present study is framed as an exploratory pilot study rather than an efficacy trial.

Despite these limitations, the study provides early-phase information regarding practical application, short-term tolerability, and microbiome characterization in a well-defined group of patients with peri-implantitis presenting with persistent suppuration. The expanded microbiome reporting also provides a more transparent description of the sequencing and analytical workflow and documents that multiple complementary analyses did not detect a major compositional change over 7 days. These observations may inform the design of future randomized controlled studies but should not be used to justify routine clinical adoption of topical L8020 therapy.

## Conclusion

Topical application of Lactobacillus rhamnosus KO3 (L8020) following standardized saline irrigation was feasible and well tolerated during the 7-day observation period. Although suppuration was absent in 7 of 10 implants at Day 7, the uncontrolled design does not permit this finding to be attributed specifically to L8020, and the change may represent transient remission related to irrigation, local manipulation, biofilm disruption, spontaneous fluctuation, or their combined effects. Expanded microbiome analyses, including Bray-Curtis beta-diversity testing, genus- and species-level taxonomic profiling, and LEfSe differential abundance analysis, did not detect substantial compositional restructuring over 7 days. The present findings do not support routine clinical implementation of topical L8020 therapy. Because incremental benefit over irrigation alone and formal cost-effectiveness were not evaluated, randomized controlled trials with appropriate comparators, larger samples, and longer follow-up are required before clinical benefit can be established.

## Data Availability

The raw 16S rRNA gene sequencing data generated in this study have been deposited in the DNA Data Bank of Japan (DDBJ) Sequence Read Archive under BioProject accession number PRJDB12664 and Run accession numbers DRR1206388-DRR1206407. The BioProject record is available at https://ddbj.nig.ac.jp/search/entry/bioproject/PRJDB12664. Other data supporting the findings of this study are available from the corresponding author upon reasonable request, subject to applicable ethical and privacy restrictions.
