# Supplementary material for: Short-term clinical observations following local L8020 application in peri-implantitis with suppuration: a pilot study
Source: Front Oral Health. 2026 Oct 20;7:1967259. doi: 10.3389/froh.2026.1967259 (PMC13727552; doi:10.3389/froh.2026.1967259)
Supplement: Supplementary file 1 [file Table1.pdf]

Supplementary Table S1. Summary of exploratory microbiome statistical analyses

Analysis	Metric / level	Baseline	Day 7 / statistic
Alpha diversity	Chao1	93.45 [73.26–140.51]	84.90 [69.75–98.56]; W = 21; p = 0.557
Alpha diversity	Faith's PD	8.86 [7.93–9.89]	7.91 [7.33–10.03]; W = 21; p = 0.557
Alpha diversity	Observed features	93.45 [73.25–140.50]	84.90 [69.75–98.50]; W = 21; p = 0.557
Alpha diversity	Pielou evenness	0.685 [0.652–0.710]	0.677 [0.648–0.701]; W = 23; p = 0.695
Alpha diversity	Shannon	4.233 [4.068–4.942]	4.210 [4.012–4.500]; W = 24; p = 0.770
Beta diversity	Bray–Curtis paired PERMANOVA	pseudo-F = 0.158	exact p = 0.883; 1,024 within-participant permutations
Differential abundance	Genus level	120 genera tested	Paired Wilcoxon + BH FDR: no significant genera (minimum q = 1.000)
Differential abundance	Species level	290 species tested	Paired Wilcoxon + BH FDR: no significant species (minimum q = 1.000)
Complementary analysis	LEfSe, phylum–species	LEfSe v1.0.8	No significant taxa detected at any taxonomic level

Note: Alpha-diversity values are median [interquartile range] at 34,677 reads, the highest rarefaction depth available for all 20 samples, after averaging 10 rarefaction iterations per sample. Baseline and Day 7 were compared using two-sided paired Wilcoxon signed-rank tests. BH FDR, Benjamini–Hochberg false-discovery-rate correction.
